# Passive Smoking Indicators in Italy: Does the Gross Domestic Product Matter?

**DOI:** 10.3390/ijerph15092045

**Published:** 2018-09-18

**Authors:** Giuseppe La Torre, Cristina Sestili, Rosario Andrea Cocchiara, Sara Cianfanelli, Lorenza Lia, Alice Mannocci

**Affiliations:** Department of Public Health and Infectious Diseases, Sapienza University of Rome, Piazzale Aldo Moro 5, 00185 Rome, Italy; cristina.sestili@uniroma1.it (C.S.); rosario.cocchiara@uniroma1.it (R.A.C.); sara.cianfanelli@uniroma1.it (S.C.); lorenza.lia@uniroma1.it (L.L.); alice.mannocci@uniroma1.it (A.M.)

**Keywords:** passive smoking, Italy, Gross Domestic Product

## Abstract

Background: The aim of this study is to analyse the correlation between regional values of Gross Domestic Product (GDP) and passive smoking in Italy. Methods: The outcome measures were smoking ban respect in public places, workplaces and at home, derived from the PASSI surveillance for the period 2011–2017. The explanatory variable was GDP per capita. The statistical analysis was carried out using bivariate and linear regression analyses, taking into consideration two different periods, Years 2011–2014 and 2014–2017. Results: GDP is showed to be positively correlated with smoking ban respect in public places (*r* = 0.779 *p* < 0.001; *r* = 0.723 *p* < 0.001 in the two periods, respectively), as well as smoking ban respect in the workplace (*r* = 0.662 *p* = 0.001; *r* = 0.603 *p* = 0.004) and no smoking at home adherence (*r* = 0.424 *p* = 0.056; *r* = 0.362 *p* = 0.107). In multiple linear regression GDP is significantly associated to smoking ban respect in public places (adjusted β = 0.730 *p* < 0.001; β = 0.698 *p* < 0.001 in the two periods, respectively), smoking ban in workplaces (adjusted β = 0.525 *p* = 0.020; β = 0.570 *p* = 0.009) and no smoking at home (adjusted β = 0.332 *p* = 0.070; β = 0.362 *p* = 0.052). Conclusions: Smoking ban is more respected in Regions with higher GDP. For a better health promotion, systematic vigilance and sanctions should be maintained and strengthened, particularly in regions with low compliance with smoking bans.

## 1. Introduction

Tobacco smoke is the major cause of premature deaths due to several diseases that could be prevented worldwide [1,2].

Second-hand smoke (SHS), known also as ‘environmental tobacco smoke’, ‘passive smoking’ or ‘involuntary smoking’ [3], is a phenomenon inherently derived from tobacco smoke, potentially present in all places such as at home, in the workplace and in public places. It consists of smoke released from the burning of tobacco products between the sidestream smoke (SM), which is the amount of smoke that spreads throughout the air from the lighted end without being inhaled by the smokers, and the mainstream smoke (MS), the smoke breathed out by the smoker. Sidestream smoke and mainstream smoke have similar constituents but in different quantities, the ratios of sidestream to mainstream smoke differ widely depending on the considered component [4].

At the physical-chemical level there are no particular differences between active and passive smoking; the only differences are the combustion temperature and the percentage of oxygen available (higher for active smoke). In both cases there are about 4000 different chemicals, carcinogens (polycyclic hydrocarbons, benzene, nitrosamines), irritants and allergenic substances such as formaldehyde, harmful gases such as carbon monoxide or irritants such as sulfur and nitrogen oxides, in addition to nicotine. In enclosed spaces cigarette smoke can create very high concentrations of fine dust, up to 100 times higher than the legal limits allowed for the external environment and prolonged indoor exposure to the 4000 substances present in the smoke can represent a source of pollution higher than the atmospheric of the most polluted metropolises [5].

Secondhand exposure to tobacco smoke causes cardiovascular diseases and lung cancer in nonsmoking adults and sudden infant death syndrome, acute respiratory infections, middle ear disease, exacerbation of asthma and decreased lung function in children [6]. According to the data of the Centers for Disease Control and Prevention (CDC) there is no safe threshold of secondhand smoke exposure, so short exposure can also threaten population’s health. Since 1964, in USA approximately 2,500,000 nonsmokers have died from diseases caused by exposure to secondhand smoke [6].

Indicators measuring exposure to second-hand tobacco smoke can be direct, indirect and surrogate. It seems difficult to have extensively direct data of SHS exposure of a population. In 1998, the CDC and WHO developed the Global Youth Tobacco Survey (GYTS) included in the Global Tobacco Surveillance System (GTSS); it reports the widest collection of data of secondhand smoke exposure in children, based on national school surveys [7]. In Europe the major source of data on SHS exposure is provided by Eurobarometer surveys that collect information from 2006 to 2017 in all UE countries.

According to the Eurobarometer survey published in March 2017, with regard to SHS exposure in public places, a fifth of respondents declare that they have come across people smoking inside bars, with a decrease of 5% since the survey of 2015. However, this report shows a huge difference among European countries, with high rates of SHS exposure in drinking establishments in countries of Southern Europe such as Greece, where the response of SHS exposure in places such as bars has increased to 87%, Croatia with 77% of the respondents, and Czech Republic with 73%. Concerning the eating establishments such as restaurants, the proportion of respondents that observed indoor smoke is lower, namely 9%. In these settings the rates differ less among countries; nevertheless, Greece shows a high rate (78% of respondents) and Cyprus 51% [8].

Due to the difficulties to collect direct measures of SHS exposure in each country, the prevalence of smoking in male and female population can be considered as surrogate measure of passive smoking exposure [9].

The knowledge of adverse health effects and the considerable costs of associated treatment for passive smoke led to many countries banning smoking in workplaces and in indoor and outdoor public places. Nevertheless, only 20% of the world’s population is protected by national anti-smoking bans. In many countries there is no smoking ban intervention yet or they are not reported, especially in Africa (i.e., Burundi, South Sudan, Sierra Leone). There are also countries where laws are applied only partially or in a limited way (South-East Asia Region) and exposure to passive smoke remains a serious problem [10]. 

In line with the trend of the prevalence distribution, it can be speculated that future diseases caused by tobacco smoke will concern mainly people in worse social and economic conditions, increasing health inequalities [2]. In Italy the tobacco prevalence discloses relevant differences among regions, and the decreasing trend is less prominent in Southern regions. According to Gallus et al. in 2010, 21.7% of Italians were current smokers, including 23.9% of men and 19.7% of women. The prevalence in men was always higher than in women, but for the first time, the prevalence in women in the middle-aged population (45–64 years old) is higher than in men, with 25.9% in women and 25.6% in men. Actually, the male and female smoking prevalence does not differ among individuals with high degrees of education and in the northern and central Italian regions [11]. Gorini et al. studied social determinants in smoking habits during the 30 years up to 2009 in Italy. According to their findings, the prevalence of male smoking dropped constantly from 56.1% in 1980 to 30.2% in 2009, despite concurrent increasing differences among different educational groups. In fact in 2009, the prevalence in poorly educated men was 53% higher than in graduates. On the other hand, female smoking rates shows a different trend, remaining firm around 18% but, as in male population, the results show a decreasing prevalence in highly educated women compared to low educated ones, in a pattern starting from North to South and from younger women to older ones. Moreover, the educational inequalities in female population from the north and central regions seem to have decreased in the last decade, in contrast with the trend in the male populatio [12].

Many studies have documented social differences in passive sqke exposure. In the countries where tobacco smoke prevalence is high, SHS exposure occurs more often among the poorest families. References [13,14,15,16,17,18] found that male sex, lower education levels, lower individual income, and living in a small rural area are associated with increased exposure to secondhand smoke in workplaces [15]. In the Chinese study by Yang et al. [19], passive smoke exposure was significantly related to Gross Domestic Product (GDP) per capita. In particular exposure was highest in cities with the lowest GDP per capita. In the study carried out by Martinez-Sanchez and coll. [20], the population of southern Italy showed more exposure to SHS, and even Minardi et al., in their paper, confirmed a higher compliance with the smoking bans in northern and central Italy compared to the south. According to them, there was an increase of respect for bans in work places (5%) and in hospitality premises (3%), and of the percentage of smoke-free homes (9%) 8 years after the smoking ban implementation, but with a significant variation from north-south [21].

In 2003 the Italian Government, as one of the first countries in Europe, approved the so-called “Sirchia Law”, which banned smoking in all indoor public places. The prohibition included all indoor public places, such as offices and restaurants [22]. The implementation of the smoking ban after the law, in January 2005, led to a considerable reduction of tobacco consumption, and consequently of passive smoke [23,24,25,26,27].

However, this evidence is based on sample populations. In Italy in 2006, the Ministry of Health established an overall health status surveillance system named PASSI (Progressi delle Aziende Sanitarie per la Salute in Italia) concerning lifestyles and behavioral risk factors associated with chronic diseases, such as tobacco smoke [28]. This surveillance made it possible to quantify the levels of compliance with smoking bans in Italy, understood as the effective application of indoor smoking bans, through interviews that investigated the respondents’ perception of compliance with bans in public places and workplaces frequented in the previous 30 days and at home. Data collected by PASSI are aggregated for the twenty-one Italian regions, providing a picture at the national level. So, the aim of this paper is to analyse the correlation between Gross Domestic Product (GDP) per capita by region and perceptions of compliance with the smoking ban in public avenues, at workplace and at home, according to data from the PASSI surveillance system.

## 2. Materials and Methods

The outcome measures indicating passive smoking in different settings were, at the regional level, the prevalence of:

- Perception of compliance with the smoking ban at public places;
- Perception of compliance with the smoking ban in the workplaces;
- No smoking at home.

The source of these measures were data for the periods 2011–2014 and 2014–2017 that were retrieved from the PASSI surveillance system. This project, by administering surveys, continuously collects information from the Italian adult population (18–69 years) concerning smoking habits, physical inactivity, excess weight, alcohol consumption, diet, cardiovascular risk, access to cancer screening and the quality of life connected to health [28].

The main explanatory variable was GDP per capita (Eurostat 2015), also recorded at the regional level [29].

The bivariate analysis between these variables was performed using the Spearman correlation coefficient (r). The linear regression analysis (simple and multiple) was performed using three different models considering the outcome measures as dependent variables and GDP per capita as the main independent variable. In the multivariate analysis, the models were adjusted for the total population of the Region and its male percentage [30]. The results of the linear regression analysis are presented as standardized beta coefficient and p-value. The goodness of fit of the models was assessed using R^2^.

The statistical significance was set at *p* < 0.05. The analysis was carried out using IBM-SPSS, release 25.0 for Windows.

## 3. Results

A descriptive analysis of the GDP distribution among 21 Italian Regions was performed. Data concerning year 2015 (Eurostat), showed a GDP mean value of 26,714.29 €, with a SD of 7256.6. The rate of ban respect through each region was assessed, considering ban in public places, in workplaces and no smoking at home between year 2011–2014 and year 2014–2017. Table 1 shows the rates of adherence to each category [21].

The bivariate analysis concerning the association between GDP and smoking ban respect in public places showed a correlation coefficient of 0.779 (*p* ≤ 0.001) concerning the period 2011–2014 and 0.723 (*p* ≤ 0.001) for the years 2014–2017.

Furthermore, the analysis of the association between GDP and smoking ban respect in the workplace showed also a significant correlation coefficient of *r* = 0.662 (*p* = 0.001) for the years 2011–2014, and *r* = 0.603 (*p* = 0.004) concerning years 2014–2017.

Adherence to no smoking at home showed that the correlation coefficient was not statistically significant considering the years 2011–2014 (0.424; *p* = 0.056) and 2014–2017 (0.362; *p* = 0.107). In Table 2 the results of the bivariate analysis are presented.

The linear regression analysis (Table 3) confirmed the results from the bivariate analysis.

The simple linear regression model considering smoking ban compliance in public places showed a beta coefficient for GDP per capita of 0.735 (*p* < 0.001) considering the years 2011–2014 and of 0.710 (*p* < 0.001) considering the years 2014–2017. The multiple regression model showed a beta coefficient of 0.730 (*p* < 0.001) considering the years 2011–2014 and of 0.698 (*p* < 0.001) considering the years 2014–2017.

The simple linear regression model considering smoking ban respect in workplace showed a beta coefficient of 0.549 (*p* = 0.01) concerning the years 2011–2014 and of 0.597 (*p* < 0.001) concerning the years 2014–2017. The multiple regression model showed a beta coefficient of 0.525 (*p* = 0.020) and 0.570 (*p* = 0.009) respectively concerning the years 2011–2014 and 2014–2017.

The simple linear regression model considering no smoking at home showed a beta coefficient of 0.427 (*p* = 0.054) considering years 2011–2014 and of 0.446 (*p* = 0.043) considering years 2014–2017. The multiple regression model showed a beta coefficient of 0.332 (*p* = 0.070) and 0.362 (*p* = 0.052) respectively for years 2011–2014 and 2014–2017.

## 4. Discussion

Passive smoking still represents one of the most important and most widespread health threatening forms of exposure. According to the PASSI Surveillance data, in Italy around 9 adults out of 10 stated that the ban of smoking in public places and in workplaces, in the thirty days prior to interview, is always or almost always respected (Table 1). However, there are clear regional differences and a clear North-South contrast, with compliance with the smoking ban s less likely to occur the southern regions.

GDP per capita is considered an indicator of the standard of living. In Italy GDP is lower in southern regions where socio-economic indicators (high unemployment rate and low educational level) are less favourable. This study reveals that regions with higher GDP per capita have higher percentages of compliance with smoking bans. GDP per capita is significantly related to respect for the smoking ban in public places and in workplaces, but this correlation is not significant for smoking restrictions at home. GDP in fact becomes a parameter that describes the cultural awareness of the individual in the commitment to protect the health of the community in which he lives. It should also be noted that the concomitance of various economic and social factors contributes in determining the correlation between wealth and compliance with regulatory obligations.

These findings are consistent with the results of the study by Yang [19]. Other researches focused on second-hand smoke and socioeconomic determinants. In the study carried out by Liao et al. [31], parents with lower education and lower annual incomes smoke more frequently in the presence of their children. Similar results about children’s passive smoke exposure were found by Kuntz et al. [32] and by Hajizadeh and Nandi [33]. Moreover, King et al. [34] and Tsai and colleagues [35] found that of SHS exposure was significantly lower among graduates and with higher annual household income.

More efforts should be realised to sensitize the population to the harmful effects of passive smoke on health. According to Martinez-Sanchez et al. [20] the lower rate of smoking restriction at home corresponds to an erroneous perception of SHS harmful effect by population. The lower rate of no smoking at home highlighted in the latest PASSI report can be linked to the absence of the type of legislation concerning private houses that can pose a deterrent in public places and in workplaces. This concern could reflect a low awareness of the dangerous risk represented by SHS for health in public opinion.

We need to acknowledge that this paper has some limitations. First of all, this is an ecological study, and the association between GDP and passive smoking indicators has to be considered with caution. Data by PASSI are collected through questionnaires, and information bias could be an issue. However, as reported by Samet and Yang [36], SHS can be assessed through indirect parameters that consist in survey conducted through questionnaires. Finally, this study analyses data from all the Italian regions and it is therefore possible that selection bias could have an impact on the considered variables within the same region. However, the PASSI surveillance has been demonstrated to be a reliable system for lifestyle data.

## 5. Conclusions

Thanks to the enforcement of the law on smoking bans, important results have been achieved but areas of action to be further strengthened remain, not only in the domestic context, but also in open spaces in the presence of minors, such as public parks, playgrounds, beaches, sports centres, and train stations, which could be regulated.

This study shows that within the national context, adherence rates to smoking bans are variable and follow the distribution of the economic wealth of the regions. It will be necessary to carry out studies to highlight the factors influencing the different adherence to legislation, and on the basis of this, implement targeted programs to increase adherence rates. Furthermore, for a better health promotion, systematic vigilance and sanctions should be maintained and strengthened, mostly whenever low compliance towards smoking bans is registered.

## Figures and Tables

**Table 1:** Rate of adherence to smoking ban in public places, workplaces and no smoking at home.

	Respect for Smoking Ban in Public Places (%)	Respect for Smoking Ban in the Workplace (%)	Adherence to No Smoking at Home (%)
2011–2014	89.7	91.4	78.7
2014–2017	91.0	93.1	81.7

**Table 2:** Results of the bivariate analysis of correlates of GDP per capita.

Variables	Correlation Coefficient	*p*-Value
Smoking ban respect in public places 2011–2014	0.779	<0.001
Smoking ban respect in the workplace 2011–2014	0.662	0.001
No smoking at home 2011–2014	0.424	0.056
Smoking ban respect in public places 2014–2017	0.723	<0.001
Smoking ban respect in the workplace 2014–2017	0.603	0.004
No smoking at home 2014–2017	0.362	0.107

**Table 3:** Results of the linear regression analysis.

Dependent Variable	GDP Per Capita Standardized Beta (*p*) *	R^2^ of Simple Linear Regression Model	GDP Per Capita Standardized Beta (*p*) ^	R^2^ of Multiple Linear Regression Model
Smoking ban respect in public places 2011–2014	0.735 (<0.001)	0.540	0.730 (<0.001)	0.579
Smoking ban respect in public places 2014–2017	0.710 (<0.001)	0.504	0.698 (<0.001)	0.544
Smoking ban respect in the workplace 2011–2014	0.549 (0.01)	0.301	0.525 (0.020)	0.318
Smoking ban respect in the workplace 2014–2017	0.597 (<0.001)	0.356	0.570 (0.009)	0.379
No smoking at home 2011–2014	0.427 (0.054)	0.182	0.332 (0.070)	0.517
No smoking at home 2014–2017	0.446 (0.043)	0.199	0.362 (0.052)	0.508

* simple linear regression model; ^ multiple linear regression model, adjusted for male percentage and regional population.
